# Preparation of silver colloids with improved uniformity and stable surface-enhanced Raman scattering

**DOI:** 10.1186/s11671-015-0746-1

**Published:** 2015-02-05

**Authors:** Wei Meng, Fang Hu, Xiaohong Jiang, Lude Lu

**Affiliations:** School of Sciences, China Pharmaceutical University, Nanjing, 211198 China; Key Laboratory for Soft Chemistry and Functional Materials of Ministry Education, Nanjing University of Science and Technology, Nanjing, 210094 China

**Keywords:** Silver colloids, SERS, Uniformity, Crystal violet

## Abstract

Silver colloids of uniform shape and size are prepared by a two-step reduction. Small silver particles form initially by the rapid reduction of silver nitrate with sodium citrate at 100°C and then grow at 92°C. The reaction processes and resulting silver colloids are characterized by transmission electron microscopy, ultraviolet–visible absorption spectrophotometry, zeta-potential measurements, and Ag^+^ concentration analysis. The surface-enhanced Raman scattering (SERS) activity of the silver colloids is then investigated, using crystal violet (CV) as a SERS probe. The silver colloids exhibit uniform shape and size and stable SERS activity. The average size of the silver particles is 47 nm (14% relative standard deviation), while the average sizes of the silver colloids prepared at 100°C and 92°C are 41 (30%) and 71 nm (33%), respectively.

## Background

Surface-enhanced Raman scattering (SERS) was first observed from pyridine adsorbed on a roughened silver electrode by Fleischmann et al. [1]. SERS has since attracted much attention because of its high sensitivity to low analyte concentrations. The sensitivity enhancement factor is on the order of 10^5^ to 10^6^ but can reach 10^14^ to 10^15^ [2]. Previous SERS studies of bulk samples have elucidated the SERS mechanisms and shown that electromagnetic and chemical enhancements operate simultaneously. The former is due to surface plasmon resonance (SPR) confined to the near-surface region, while the latter is due to surface active sites or charge transfer between the adsorbate and substrate [3,4]. Regardless of electromagnetic or chemical enhancement, active substrates and adsorbed analytes (mono- or multi-layers) are both necessary for producing high-quality SERS spectra. Substrate activity depends on the scale of its features (10 to 100 nm) and its morphology [5-7].

Many SERS-compatible active substrates have been investigated. Ag and Au are the most widely studied, because of their high enhancement factors, but transition metals, semiconductors, and other materials have also been studied [8-10]. The morphologies of SERS substrates can include roughened electrodes, colloids, island films, and nanoarrays. Silver and gold colloids, especially citrate-reduced colloids, are the most commonly used SERS substrates because they are easily prepared and can be well characterized. The shape and size of their particles can also be controlled by inexpensive, versatile approaches.

Stable gold colloids with narrow size distributions were recently synthesized by a reduction with citrate [11]. Improved shape/size control of silver particles - along with the preparation of uniform, stable silver colloids - has become an active research area. Citrate-reduced silver colloids can be prepared by reducing silver nitrate with sodium citrate at its boiling point, according to Lee and Meisel [12], wherein citrate also stabilizes the dispersion. In contrast to gold colloids, silver colloids can exist as prisms, rods, and disks of varying size, though spherical particles are the most common. Methods for controlling particle morphologies include adding stabilizers, adjusting heating and stirring rates, changing the citrate/silver nitrate ratio, and adjusting the pH [13,14]. Stable, monodisperse sols can be obtained by such methods. However, some added substances decrease the SERS activity of silver particles in substrates used for diagnosing and characterizing analytes. This is attributable to competitive adsorption of the analyte and added substance. Thus, understanding the nucleation mechanism of silver colloidal particles is important for improving size control.

Probe molecules are used to investigate the SERS activity of substrates and usually contain azo, carboxyl, or thiol groups. These analytes strongly adsorb to a substrate’s surface, which maximizes SERS enhancement. Crystal violet (CV) exhibits SERS activity on silver surfaces [15,16].

In the current study, silver colloids are prepared by a two-step reduction of silver nitrate at high (100°C) and low (92°C) temperatures in the presence of sodium citrate. These uniformly sized silver particles have an average size of 47 nm and a relative standard deviation of 14%. CV is used as a SERS probe, and the silver particles exhibit high SERS activity compared with the colloids prepared at 100°C or 92°C.

## Methods

Polyvinyl alcohol (PVA), CV (C_25_H_30_ClN_3_), silver nitrate (AgNO_3_), sodium citrate (Na_3_C_6_H_5_O_7_ · 2H_2_O), 2,4,5,7-tetrabromofluorescein (TBF), and 1,10-phenanthroline (PHEN) were all of analytical grade and were obtained from Sinopharm Chemical Reagent Co., Ltd. Ultraviolet–visible (UV–vis) absorption spectra were recorded on a TU-1810 spectrophotometer (Beijing Purkinje General Instrument, Beijing, China), using 2 mm-path-length quartz cells. Aliquots of the reaction solutions were removed at different times and cooled immediately in ice water to prevent further reaction. Measurements were taken from undiluted samples. Zeta potentials were measured using a Malvern Zetasizer 90 instrument. Samples for Raman spectroscopy measurements were prepared by adding 1 × 10^−4^ mol L^−1^ CV solution to the silver colloids. Samples were incubated for 30 min, and Raman spectra were recorded on a Renishaw Micro-Raman spectroscopy system and averaged over seven measurements. Excitation was provided by an Ar ion laser (514 nm). A 50×, 0.50 NA Leica objective lens with a long working distance was used to focus the laser on the surface of the silver colloid samples. Excitation power was 20 mW, integration time was 10 s, and the calculated spot size was about 1.25 μm. A droplet of silver colloid suspension was allowed to dry on a copper grid for transmission electron microscopy (TEM) observations. TEM images were collected using a Tecnai 12 (Philips) microscope, operated at 120 kV.

### Preparation of samples

Sample A was prepared as follows: 194 mL of distilled water in a three-neck flask was heated to 100°C under reflux with vigorous stirring. Upon boiling, 2 mL of 0.1 mol L^−1^ AgNO_3_ and 4 mL of 1% sodium citrate were added successively to start the reaction. The solution was refluxed for 40 min then allowed to cool to room temperature. Sample B was prepared similarly, except the temperature was 92°C and the solution was refluxed for 100 min. Sample C was prepared by a two-step reduction: 2 mL of 0.1 mol L^−1^AgNO_3_ and 4 mL of 1% sodium citrate were added to 170 mL of boiling distilled water under reflux, and the reaction was allowed to continue for 4 min at 100°C. The reaction vessel was then transferred to a water bath at 92°C and allowed to remain there for 80 min to continue the reduction of silver nitrate. Upon transfer, the temperature of the reaction mixture was quickly reduced to 92°C by adding cold water. The three silver colloid samples were all adjusted to a final volume of 200 mL. All solutions were continuously stirred during preparation. All glassware was cleaned with dilute nitric acid and thoroughly rinsed with distilled water before use.

### Determination of Ag^+^

The modified spectrophotometric determination of Ag^+^ was based on a method developed by Mohamed and Roland [17]. A stock solution was prepared by adding 25 mL of 5 × 10^−4^ mol L^−1^ PHEN, 0.6 mL of 2 × 10^−3^ mol L^−1^ TBF, and 40 mL of 0.1% PVA into a 100 mL flask and diluting with water to produce a volume of 100 mL. Then, 10 to 35 μL of 1 × 10^−3^ mol L^−1^ silver nitrate was added to 5 mL of this stock solution. The characteristic absorbance of the ternary [PHEN-Ag-PHEN]_2_TBF complex at 548 nm was recorded in the UV–vis absorption spectrum, using 1 cm-path-length quartz cells. PVA is a stabilizer for the ternary [PHEN-Ag-PHEN]_2_TBF complex and can improve its solubility and analytical sensitivity. The results from several samples showed that the maximum absorbance at 548 nm increased linearly with increasing Ag^+^ concentration in the range of 2 × 10^−6^ to 7 × 10^−6^ mol L^−1^. A regression equation of *A* = 50, 310*c* (mol L^−1^) was obtained, where *A* indicates maximum absorbance and *c* the Ag^+^ concentration. A 10 to 30 μL aliquot of reaction solution was added to 5 mL of stock solution to determine Ag^+^ concentration. The silver-particle absorbance at 548 nm was <0.01 for each sample, so centrifugation was not necessary.

## Results and discussion

The widely used method of preparing silver colloids by reduction with sodium citrate was first proposed by Lee and Meisel [12]. Different theories for the silver-particle-formation mechanism have excited much debate, a comprehensive study on which was performed by Munro et al. [18]. Spectroscopic results for UV–vis absorption and photon correlation suggest that the initial reduction of Ag^+^ occurs within 2 min of adding citrate, (forming the initial 60- to 80-nm-diameter particles). Subsequent heating disperses the large particles, yielding a semi-monodisperse silver colloid with a mean particle diameter of 27 nm. The maximum absorption wavelength (*λ*_max_) blueshifts, while the maximum absorbance increases during dispersion. Alternative colloid-formation mechanisms have also been proposed: Pillai and Kamat [19] suggested that only a few silver seed crystals form during the initial citrate-reduction procedure. In this framework, the seeds strongly complex with citrate anions and then grow into larger particles until electrostatic repulsion prevents further aggregation. Larger particles continue to grow at the expense of smaller ones, via Ostwald ripening.

These details of the silver-particle-formation mechanism suggest that heating, stirring, the time and rate of reagent addition, and reagent concentrations should affect particle formation. Different mechanisms may occur under different reaction conditions. Aggregation, dispersion, nucleation, seed formation, and crystal growth may coexist (and be balanced) in the reaction procedure. Dong and Yang [14] controlled the nucleation and growth of silver particles by adjusting the pH.

The formation of silver particles is often investigated using UV–vis absorption spectroscopy, which does not always provide a comprehensive understanding of the formation mechanism. Crystallization consists of nucleation, seed crystal formation, and crystal growth. Nucleation involves the clustering of molecules of a critical size, which comprise nuclei. Once these nuclei form, they quickly grow into seed crystals, which trigger the crystal-growth process. Nucleation remains difficult to observe, even with current TEM technology. Nevertheless, TEM and other methods suitable for characterizing Ag^+^ should be used to more clearly understand the formation of silver particles.

Many published studies have addressed Ag^+^ detection by means such as UV–vis absorption and fluorescence spectroscopies and electrodes with Ag^+^ selectivity. Other more complex spectrometric techniques involve flame and electrothermal atomic adsorption spectrometry (FAAS and ETAAS) as well as inductively coupled plasma mass spectrometry.

Detection of Ag^+^ in silver colloids is difficult because of its low concentration and matrix effects. Some Ag^+^ adsorbs on the silver particles. The adsorption of ions and subsequent disturbance of Ag atoms mean that the Ag^+^ concentration cannot be readily determined using selective electrodes or atomic spectroscopy, unless the Ag^+^ and Ag particles are separated. Thus, in this study, we employ a modified UV–vis spectrometric process to determine the aqueous Ag^+^ content.

**Figure 1:**
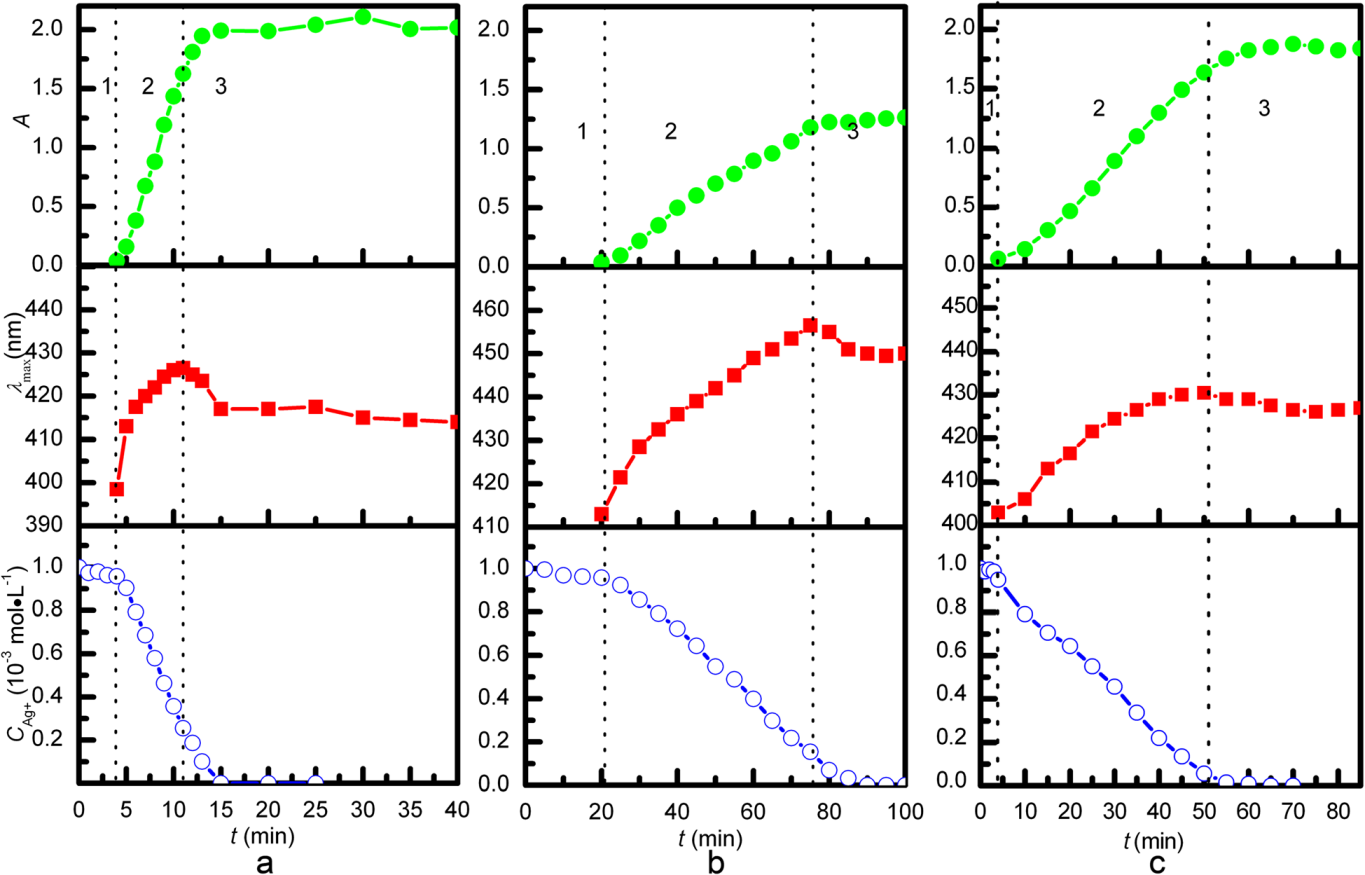
**Absorbance, λ**_**max**_ **, and Ag** ^**+**^ **concentration for the reactions: (a) 100°C; (b) 92°C; (c) two-step reduction (100°C to 92°C).**

**Figure 2:**
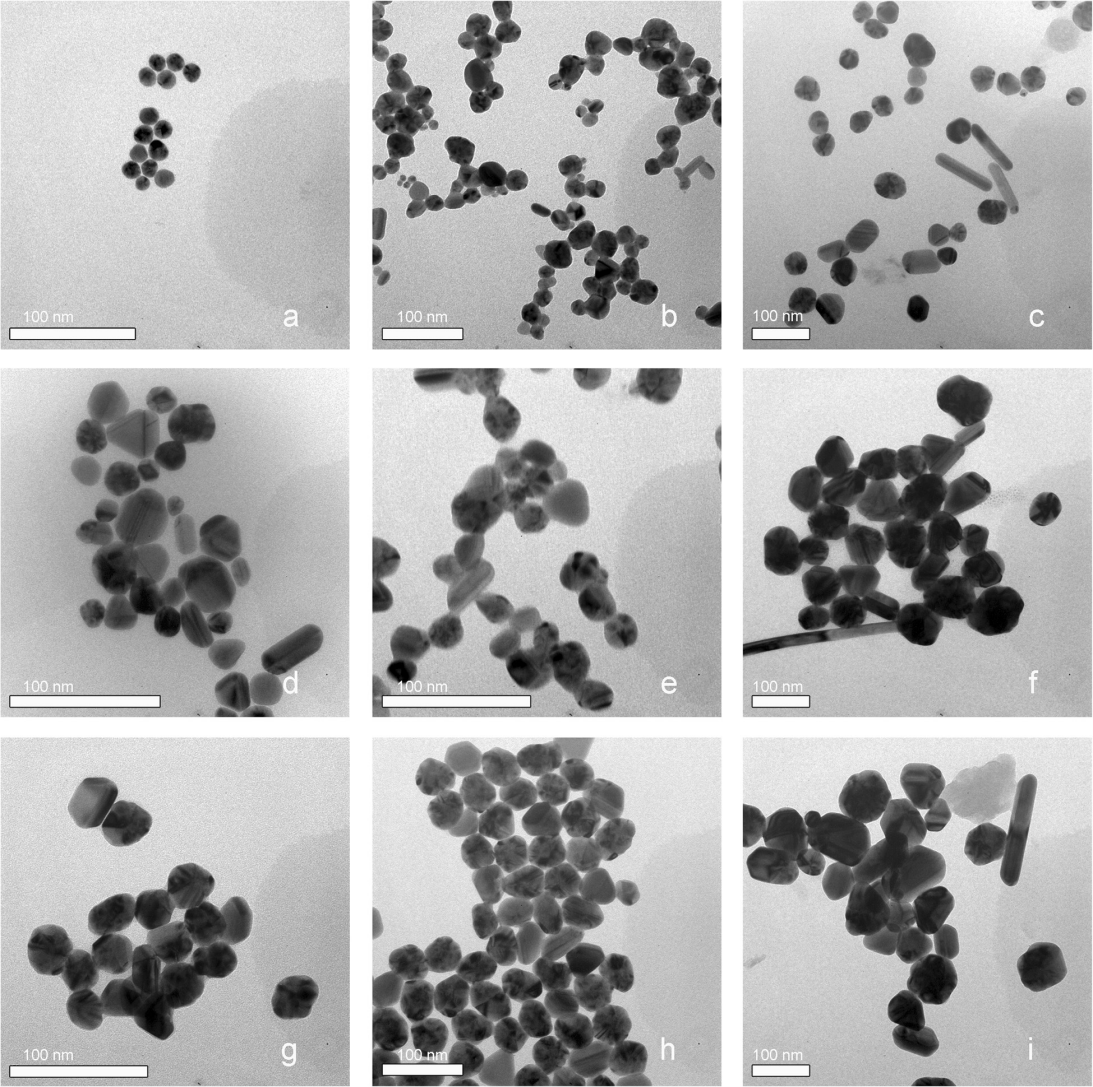
**TEM images of reaction aliquots: (a) sample A at stage 1; (b) sample A at stage 2; (c) sample A at stage 3; (d) sample B at stage 1; (e) sample B at stage 2; (f) sample B at stage 3; (g) sample C at stage 2; (h) sample C at stage 3; (i) sample D at stage 3.**

The time evolution of *λ*_max_, absorption at *λ*_max_, and Ag^+^ concentration for sample A are shown in Figure 1a. The reaction can be divided into three stages: nucleation of silver and formation of silver seeds (1), growth of silver seeds (2), and dispersion of silver particles (3). During stage 1 (0 to 4 min), the solution is initially colorless with little absorption and then becomes faintly yellow and weakly absorbs at 398.5 nm. About 5% of Ag^+^ is reduced in the first 4 min. TEM images show that the size of the silver seeds is approximately 10 nm at the end of stage 1 (Figure 2a). During stage 2 (4 to 11 min), the reduction of Ag^+^ accelerates because of catalysis by the silver particles [20]. The rapid increase in *λ*_max_ and absorbance at *λ*_max_ indicates the growth of silver seeds, which makes the solution turn gray yellow. It is difficult to exclusively restrict growth to molecular addition during stage 2, as evidenced by the particles (approximately 10 nm, see Figure 2c) existing in the final silver colloids. During stage 2, silver nuclei continue to form, creating more silver seeds, which grow by molecular addition, as shown by TEM (Figure 2b). Large and small silver particles form simultaneously, so stage 2 does not solely involve the growth of silver seeds. It should instead be seen as a continuous formation-and-growth stage for silver seeds. At 11 min, the *λ*_max_ blue shifts, and the reaction begins stage 3. About 20% Ag^+^ remains at this point. Ag^+^ continues to reduce as stage 3 progresses: *λ*_max_ shifts from 426 to 413 nm, and the absorbance intensity increases. This indicates that some silver particles aggregate into larger particles during stage 2, and that dispersion dominates stage 3. Figure 2c shows that the product is a mixture of quasi-spherical and rod-like particles [21] with an average size and relative standard deviation of 41 nm and 30%, respectively. These sizes are calculated from average values of the long and short axes of >100 particles. The continuous formation of silver seeds during stage 2 yields the wide distribution of shapes and sizes observed in the final product.

Sample B is prepared by reduction at low temperature to prevent continuous seed formation. During stage 1 (0 to 20 min) the solution is colorless and does not absorb (Figure 1b). The longer duration of stage 1 compared with sample A (100°C) is attributed to the slower reaction at 92°C. During stage 2 (20 to 75 min), the *λ*_max_ and maximum absorbance increase with decreasing Ag^+^ concentration, indicating the growth of silver seeds. In contrast to stage 2 for sample A, few silver seeds (approximately 10 nm) are observed in the TEM images of sample B during stage 2 (Figure 2e); this is attributed to the slower reaction, which hinders seed formation. Figure 2f shows that the average particle size of sample B is 71 nm, with a relative standard deviation of 33%. The lower reaction rate results in fewer seeds, which grow larger because they are subjected to the same Ag^+^ concentration as sample A. Continuous nucleation is avoided, but the particle size range is larger because of the larger size distribution of seeds (Figure 2d). Heterogeneous nucleation is the predominant nucleation type for a low Ag^+^ reduction rate and low supersaturation.

**Figure 3:**
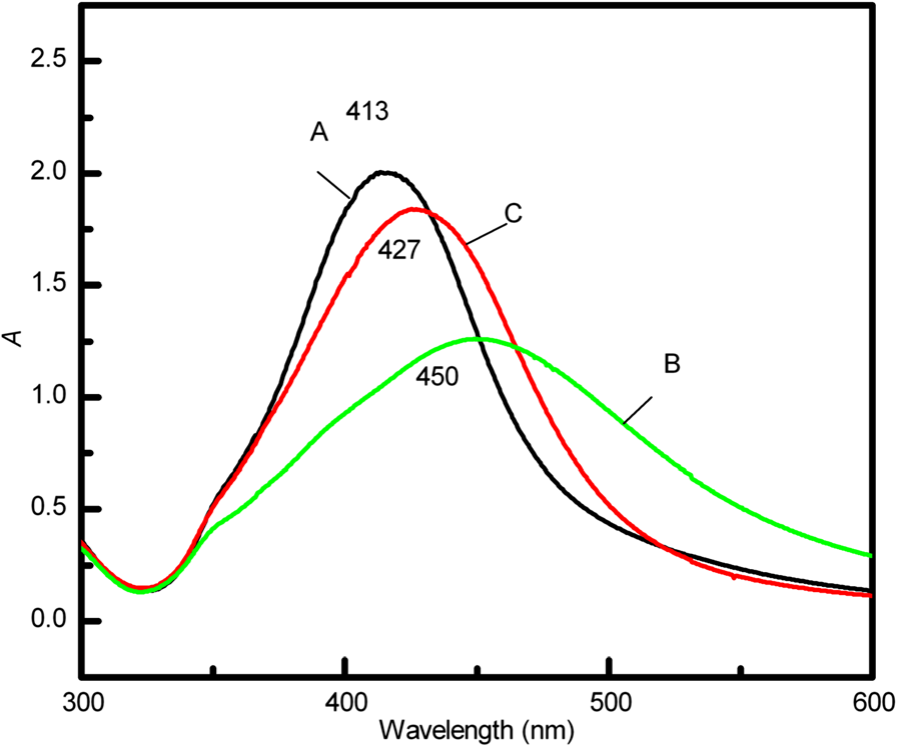
**UV–vis absorption spectra of samples A, B, and C.**

The LaMer model [22] and our previous study [23] suggest that particle size can be controlled by rapid nucleation followed by slow growth. A two-step reduction method involving a temperature change during reaction is thus carried out for sample C. After stage 1, the reaction vessel is transferred into a 92°C water bath, and the reaction mixture is quickly cooled to 92°C by adding cold water, allowing for slower growth. Figure 1c shows the change in *λ*_max_, the absorbance at *λ*_max_ in the UV–vis absorption spectrum, and the Ag^+^ concentration of sample C. (Ag^+^ is reduced more slowly at 92°C.) Under these conditions, the formation of silver seeds is forbidden (Figure 2g), and seeds grow with a depletion of Ag^+^. Thus, Ag^+^ is almost completely reduced by the end of stage 2 (4 to 50 min); *λ*_max_ occurs at 430 nm at the end of stage 2 and does not change substantially during stage 3. This suggests that growth is mainly due to molecular addition on silver-particle surfaces. Stable, quasi-spherical silver colloids are eventually obtained. The average particle size and relative standard deviation obtained from the TEM images are 47 nm and 14%, respectively (Figure 2h). The particle size of sample C is between those of samples A and B, and its relative standard deviation is lower than that of both the other samples, indicating more uniformly sized silver colloids. The peak half-width of the colloids prepared in the two-step reduction is larger than that of those prepared at 100°C (Figure 3). However, colloid quality cannot be unambiguously analyzed solely from UV–vis absorption spectra. The UV–vis absorption spectra of larger silver particles depend on particle size, because the higher-order terms contribute significantly. The plasmon bandwidth increases with increasing particle size, which is usually ascribed to extrinsic size effects because size dependence arises from the full expression of Mie’s theory [24,25]. Thus, the lower reaction temperature more effectively prevents continuous nucleation by decreasing the Ag^+^ reduction rate and supersaturation. However, sample D prepared by reaction at 100°C followed by 90°C is not uniform, since silver nanoparticles with a wide size distribution are observed in the final product (Figure 2i). Crystal growth is accompanied by dissolution. Smaller crystals and particle protrusions have higher interfacial energies and solubilities than larger crystals, which enhances the growth of bigger crystals and the uniformity of particles via Ostwald ripening. Further decreasing in temperature suppresses the oxidation of silver, making Ostwald ripening less significant. In addition, decreasing the temperature increases the required reaction time, and the reaction glassware becomes coated with silver nanoparticles. Of course, if the ratio of silver nitrate and citrate is changed, uniform silver colloids with different average sizes of nanoparticles may be prepared through two-step synthesis.

The zeta potentials of samples A, B, and C are −40.3, −41.1, and −39.8 mV, respectively. These similar potentials arise from the adsorption of citrate and its carboxylate oxidation products [18] on the silver-particle surface. Colloidal silver particles are negatively charged and thus attract positively charged CV. The zeta potentials of samples B and C are no greater than that of sample A, despite these samples’ larger particle sizes and lower surface areas. This implies that the adsorption of citrate is saturated in all three samples.

Samples for Raman spectrometry are prepared by mixing 5 mL of silver colloid with 50 μL of 1 × 10^−4^ mol L^−1^ CV solution (with a CV concentration of 1 × 10^−6^ mol L^−1^). No significant changes in the UV–vis absorption spectra of the silver colloids are observed in the presence of CV, indicating that CV does not induce particle aggregation. SERS spectra are recorded without the addition of active substances such as halide anions. The addition of such substances tends to aggregate the particles, causing stronger SPR, and thus chemically enhancing SERS activity [26]. Nie suggested that aggregation also produces hot junctions, which can increase SERS intensity [27]. This phenomenon was not observed in the present study.

**Figure 4:**
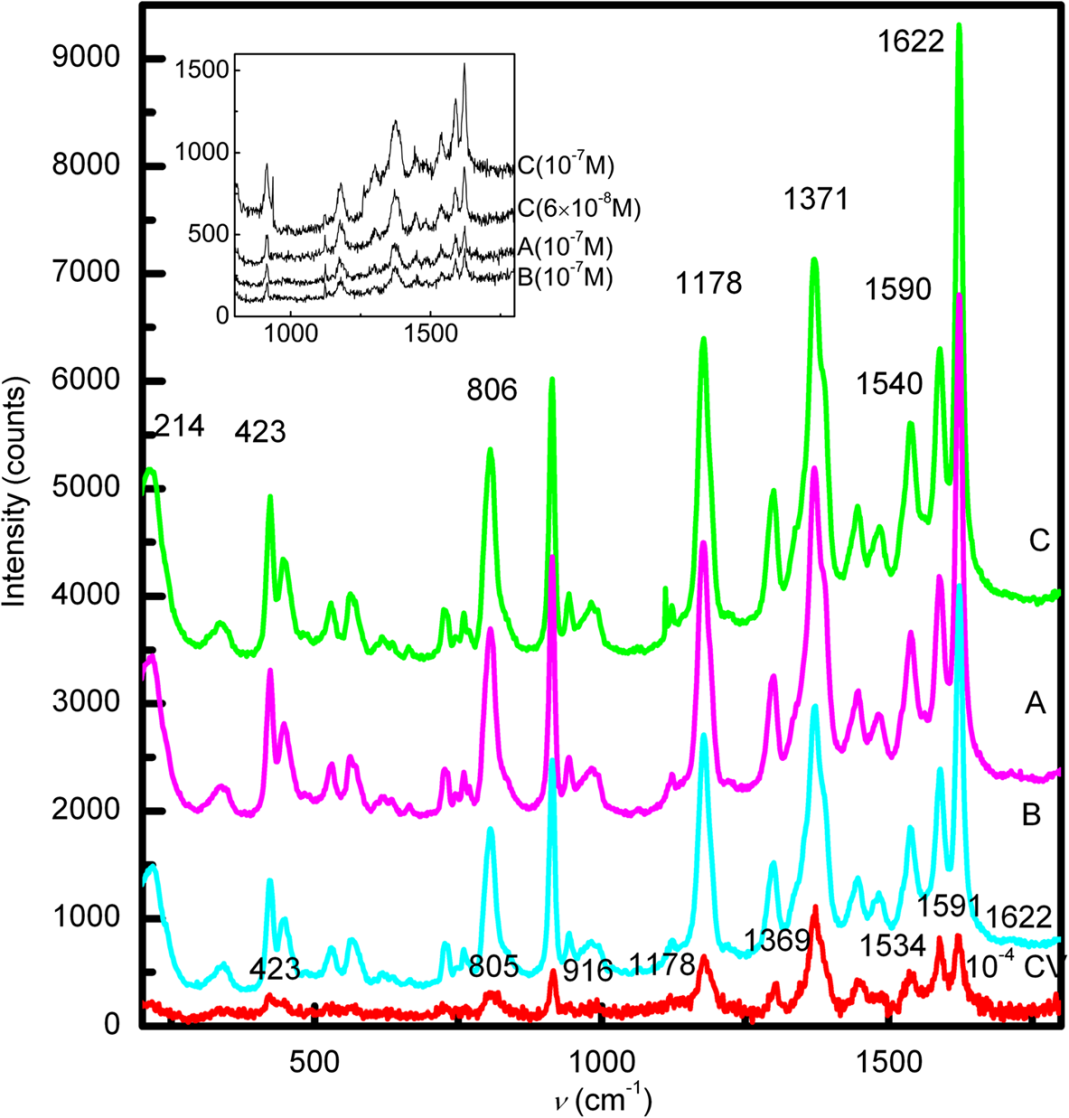
**SERS spectra of crystal violet in samples A, B, and C and Raman spectrum of crystal violet.**

**Figure 5:**
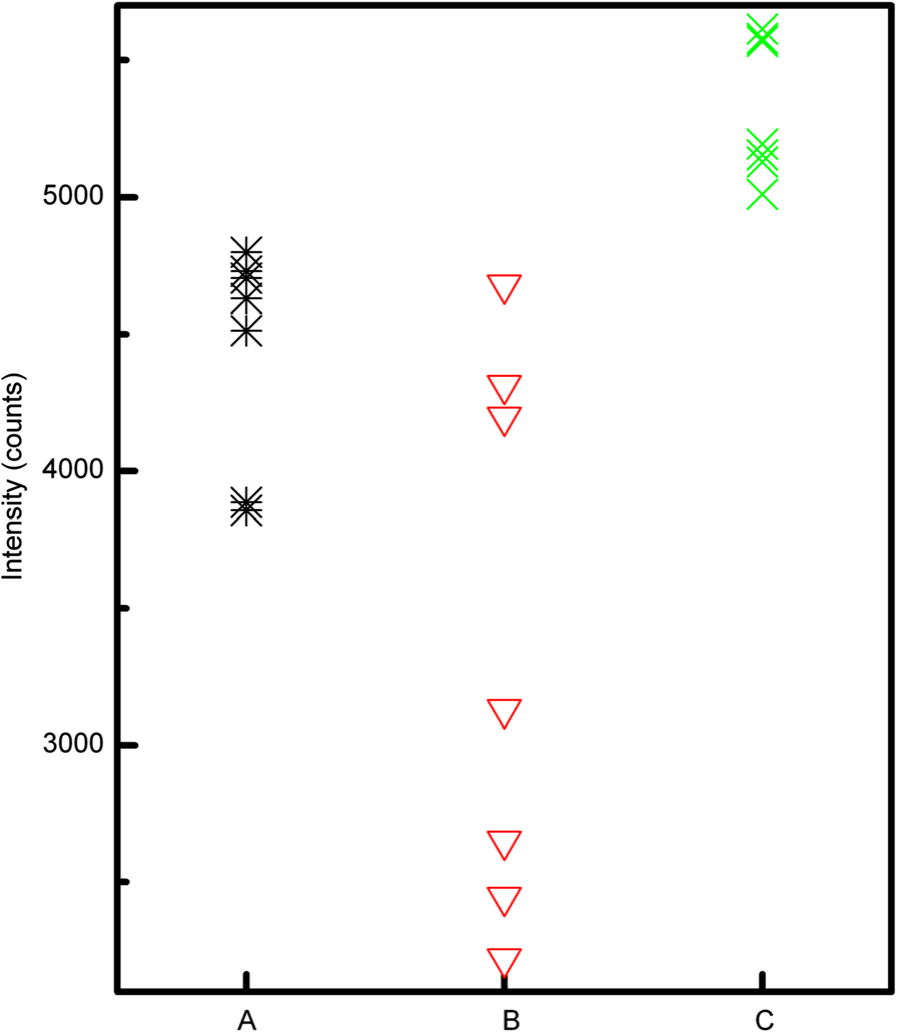
**Peak intensity at 1,622 cm** ^**−1**^ **versus measurement repetition for samples A, B, and C.**

Figure 4 shows the averaged SERS spectra of CV in samples A, B, and C, as well as the Raman spectrum of 1 × 10^−4^ mol L^−1^ CV. The SERS spectra of the three samples are of very high quality, even for 1 × 10^−7^ mol L^−1^ CV. In contrast with samples A and B, a lower concentration of CV for sample C was detected (6 × 10^−8^ mol L^−1^) (Figure 4 inset), but the normal Raman spectra of CV is not distinguished below concentrations of 10^−4^ mol L^−1^ in our experimental conditions. No significant differences exist in the spectral profiles, though the peak intensities increase in the following sequence: B < A < C. The SERS spectrum of CV contains peaks at 1,622 and 1,590 cm^−1^, corresponding to the C–C stretching vibration of the phenyl ring. The peak at 1,371 cm^−1^ is ascribed to the C–C_center_ stretching vibration, and those at 1,178 and 806 cm^−1^ are ascribed to C–H bending vibrations. The radical-ring skeletal vibration and C–N bending vibration occur at 914 and 423 cm^−1^, respectively [4,28]. Shifts in the SERS peaks are observed for all three samples, compared to the Raman spectrum of CV. This indicates chemical adsorption in all samples, as confirmed by the Ag–N stretching vibration at 214 cm^−1^ in the SERS spectra. CV likely interacts with Ag through the N lone-pair electrons [29], and SERS arises from electromagnetic and chemical enhancement. The former depends on particle size and morphology, and the latter is related to charge transfer between the adsorbate and the SERS-active substrate. Probe molecules must be adsorbed to the SERS-active substrate in either enhancement mechanism. All the current silver colloids are prepared from the same reactant concentration and thus exhibit the same adsorption force, as demonstrated by their equivalent zeta potentials. The similar particle sizes of samples A and C result in similar surface areas available to CV. The more intense SERS signals from CV in sample C may reflect its stronger SPR, because its absorption *λ*_max_ is located near the excitation wavelength. The SERS signals of CV in sample B are weaker than those in samples A and C, though its absorption *λ*_max_ approaches the excitation wavelength. Sample B has the largest particle size and lowest surface area, meaning that its surface available to CV and its number of silver particles probed by the laser are both reduced. This indicates that the amount of adsorbate also affects the SERS signal. The averaged Raman spectra, obtained by subtracting the minimum and maximum, show that sample C has the highest SERS activity. Using the CV band at 1,622 cm^−1^ as a reference, the average peak intensities of CV for sample A, sample B, and sample C are 4,428, 3,295, and 5,240 counts, respectively. The intensity of the peak at 1,622 cm^−1^ versus the measurement repetition for samples A, B, and C is shown in Figure 5; the relative standard deviation of sample C is 5% and those of sample A and B are 9% and 29%, respectively. Sample C exhibits high SERS activity and stable SERS signals, which benefits colloid applications in terms of quantitative analysis. Here, the CV band at 1,622 cm^−1^ is used as a reference to evaluate the SERS enhancement factor (*EF*), which is often estimated as:

$$ EF\kern0.5em \frac{I_{\mathrm{SERS}}/{C}_{\mathrm{SERS}}}{\theta {1}_{\mathrm{sol}}/{C}_{\mathrm{sol}}} $$

where *θ* is the fraction of adsorbed CV at equilibrium and is estimated to be 0.09 [30], *C* is the concentration of CV in the solution (*C*_sol_ 1 × 10^−4^ mol L^−1^) or in the silver colloids (*C*_SERS_ 6 × 10^−8^ mol L^−1^), and *I* is the intensity of a band of the Raman spectrum in solution (*I*_sol_) or of the SERS spectrum (*I*_SERS_). Using this expression, we obtain an estimated *EF* value of approximately 9.5 × 10^3^. Generally speaking, aggregation caused by active substances such as Cl^−1^, Br^−1^, and I^−1^ will further increase the enhancement factor by one to three orders of magnitude through the intense magnetic fields created at particle junctions, but SERS signals are not stable in this case. Thus, the actual overall *EF* can exceed 9.5 × 10^3^.

## Conclusions

Silver colloids are prepared in a two-step reduction, without capping agents. Small silver particles are generated by rapid reduction with sodium citrate at 100°C and subsequent growth at 92°C. Two other silver colloids are prepared at 100°C and 92°C for comparison. The silver colloids prepared by the two-step reduction exhibit greater uniformity in size and shape. TEM, UV–vis absorption spectroscopy, and Ag^+^ concentration measurements during reaction provided insight into the silver-particle-formation mechanism. The average particle size for the two-step reduction was 47 nm, with a 14% relative standard deviation, whereas the sizes (relative standard deviations) of particles prepared at 100°C and 92°C are 41 (30%) and 71 nm (33%), respectively. All silver colloids are negatively charged and have equivalent zeta potentials. CV is used as a SERS probe. The SERS signals of CV on the colloids had an improved stability and intensity, demonstrating the colloids’ viability for applications in quantitative SERS analysis.
